# Hyperhomocysteinemia in Patients with Polypoidal Choroidal Vasculopathy: A Case Control Study

**DOI:** 10.1371/journal.pone.0110818

**Published:** 2014-10-22

**Authors:** Hui-Chen Cheng, Jorn-Hon Liu, Shui-Mei Lee, Po-Kang Lin

**Affiliations:** 1 Department of Ophthalmology, School of Medicine, National Yang-Ming University, Taipei, Taiwan; 2 Department of Ophthalmology, Taipei Veterans General Hospital, Taipei, Taiwan; 3 Department of Ophthalmology, Cheng Hsin Rehabilitation Medical Center, Taipei, Taiwan; 4 Graduate Institute of Biomedical Electronics and Bioinformatics, National Taiwan University, Taipei, Taiwan; 5 Biomedical Electronics Translational Research Center, National Chiao Tung University, Hsinchu, Taiwan; University of Florida, United States of America

## Abstract

**Purpose:**

To determine whether elevated plasma homocysteine and serum high sensitivity C-reactive protein (hsCRP) levels, two established risk factors of vascular diseases, are associated with polypoidal choroidal vasculopathy (PCV).

**Design:**

Retrospective case-control study.

**Methods:**

One hundred and nineteen consecutive patients with PCV and 119 matched controls were enrolled in a tertiary hospital from September 2008 to June 2013. Plasma homocysteine and serum hsCRP levels were measured. Associations among plasma homocysteine, serum hsCRP levels and PCV were further evaluated using multivariable logistic regression analysis.

**Results:**

The median plasma homocysteine level was significantly higher in patients with PCV than in the controls (12.20 µmol/L vs. 9.80 µmol/L, p<0.001). The median serum hsCRP level was slightly higher in the PCV group (0.16 mg/dl vs. 0.11 mg/dl in control group, p = 0.07). After multivariable logistic regression analysis, each 1 µmol/L increase of plasma homocysteine was associated with a 1.5-fold increase in likelihood of having PCV (OR, 1.54; 95% confidence interval (CI), 1.33–1.79, p<0.001).

**Conclusions:**

Hyperhomocysteinemia was associated with PCV and might play a role in the pathogenesis of PCV.

## Introduction

Polypoidal choroidal vasculopathy (PCV), first proposed by Yannuzzi et al, [1] is characterized by an inner choroidal vascular network ending in an aneurysmal bulge, visible clinically as a reddish orange, polyp-like structure. The affected eye typically presents with multiple recurrent serous or hemorrhagic detachments of the retinal pigment epithelium (RPE) [1], [2]. The diagnostic features of PCV are the presence of subretinal nodular hyperfluorescence (the polyps), which may be associated with branching vascular networks in fundus indocyanine green angiography (ICGA) [3]. In earlier reports, PCV accounts for 20–50% of cases manifesting as exudative age-related macular degeneration (AMD) in Asia, compared to approximately 10% in patients of Western descent [2], [4]–[6]. However, PCV has been proposed as a distinct disease entity with its characteristic polypoidal structures and branching vascular networks, which is different from choroidal neovascularization (CNV) of AMD [7]. PCV tends to present at a younger age compared to exudative AMD, with a male preponderance and more frequently in black or Asian patients [1], [2], [8], [9]. Although the pathogenesis of PCV remains largely unknown, there have been several pathological reports that have noted inner choroidal vessel abnormalities with arteriosclerosis in PCV, possibly indicating an association with vascular diseases [10]–[12].

Homocysteine is a naturally occurring sulfur-containing amino acid that is produced during the metabolism of methionine. High plasma levels of homocysteine have been identified as an independent risk factor of vascular diseases, such as cardiovascular disease and stroke, dementia and Alzheimer's disease [13]–[15]. Elevated plasma homocysteine levels are also observed in patients with retinal vascular occlusion diseases, pseudoexfoliative glaucoma and diabetic retinopathy [16]–[19]. Patients with AMD may also present with elevated plasma homocysteine levels but results are inconsistent [20], [21].

C-reactive protein (CRP) is a systemic inflammatory biomarker and a risk factor of cardiovascular disease [22]. *Kikuchi et al* [23] found a significant association between elevated high sensitivity CRP (hsCRP) levels and PCV in the Japanese, and concluded that inflammatory processes were involved in the pathogenesis of PCV. However, further correlations need to be elucidated.

From the clinical manifestations and pathological reports, we propose a hypothesis that PCV is a vascular disorder related to arteriosclerosis, which may be associated with the risk factors of vascular diseases. The purpose of this study is to determine whether elevated plasma homocysteine and serum hsCRP levels, two established risk factors of vascular diseases, are associated with PCV.

## Materials and Methods

### Study subjects

The authors conducted a case-control study with retrospective recruitment of study subjects from a tertiary hospital between September 2008 and June 2013. The study protocol adhered to the tenets of the Declaration of Helsinki. It was approved by the Institutional Review Board of the Taipei Veterans General Hospital. Though the requirement for informed consent was waived by the approving IRB, verbal informed consent was obtained and recorded on the medical chart.

Patients diagnosed with PCV were recruited consecutively during the study period. The control group included subjects who were undergoing routine physical examinations or follow-up for diseases other than AMD, retinal vascular disease, diabetic retinopathy or glaucoma. Each case was then matched with one control subject on age and gender. All study subjects were of Chinese ethnicity.

Exclusion criteria included subjects without data of plasma homocysteine level, significant comorbidity that could confuse the clinical picture, such as retinal vascular occlusion, uveitis, trauma-related eye disease, other neovascular maculopathies, and media opacities preventing adequate fundus examination. Subjects with renal dysfunction, defined as having a serum creatinine level more than 1.5 mg/dl or having a history of renal failure, and a history of taking vitamin supplements, were also excluded due to their effect on plasma homocysteine [24], [25].

All participants received a complete ophthalmic examination consisting of best-corrected visual acuity, slit lamp biomicroscopy and fundoscopy. Fundus photography with fluorescein angiography (FA) taken with a fundus camera (CF-60 UD, Canon Inc., Tokyo, Japan) and ICGA (Heidelberg Retina Angiograph II, Heidelberg Engineering, Heidelberg, Germany) were performed through dilated pupils in all case subjects. The retinal images were all reviewed by two retina specialist (P.K.L. and J.H.L) in a masked fashion. Only subjects with characteristic polyp-like choroidal vessel dilatation (polypoidal lesions or polyps) on ICGA were diagnosed to have PCV [26]. The diagnosis was confirmed when both of them had come to an agreement. Location of PCV was divided into extrafoveal, juxtafoval (within 200 µm of the fovea), subfoveal and peripapillary area (within one disc diameter of the optic disc) [27].

Data including age, gender, lifestyle factors of smoking and alcohol consumption, medication histories, medical histories of hypertension, diabetes mellitus (DM), coronary artery disease, cerebrovascular event and renal dysfunction were obtained from all subjects.

### Total plasma homocysteine analysis

Fasting venous blood samples were obtained in the sitting position and collected in test tubes containing heparin. The samples were immediately centrifuged at 4°C, and the plasma homocysteine levels were measured by automated chemiluminescent immunoassay (ADVIA Centaur system, Siemens, East Walpole, MA, USA), with a sensitivity of 0.5 µmol/L and a total coefficient of variation (CV) of 6.8% at 4.9 µmol/L, 3.9% at 61.6 µmol/L. This assay correlates well with other assays, including fluorescent polarization immunoassay and high-performance liquid chromatography (HPLC) [28].

### High sensitivity C-reactive protein (hsCRP) analysis

Venous blood samples were obtained and collected in serum-separation tubes. Serum hsCRP was measured by rate nephelometry on an automated nephelometer (Immage 800, Beckman Coulter, Fullerton, CA, USA). This hsCRP assay, with an analytical sensitivity of 0.2 mg/L and a total coefficient of variation (CV) of 5.17% at 0.79 mg/L, and 3.8% at 13.4 mg/L, has been shown to correlate well with other commonly used assays [29].

Hyperhomocysteinemia and elevated hsCRP level were defined as levels above the 95th percentile of the control group.

### Statistical analysis

Continuous and categorical variables in demographic and medical characteristics were compared between PCV cases and control subjects using Student's *t*-test and Pearson's chi-square test, respectively. Plasma homocysteine and serum hsCRP levels were presented as median (interquartile range) and compared to controls using the Mann-Whitney U test because homocysteine and hsCRP levels were not normally distributed.

Multivariable logistic regression models were used to evaluate whether or not PCV was associated with plasma homocysteine or serum hsCRP. All odds ratios (ORs) were adjusted for age, gender, lifestyle factors (smoking and alcohol consumption) and medical histories (hypertension, DM, coronary artery disease, cerebrovascular event).

SPSS for Windows version 18 (SPSS Inc, Chicago, Illinois, USA) was used for these calculations. All reported P values were based on 2-sided tests and a P value of less than 0.05 was considered to be statistically significant.

## Results

One hundred and twenty-four case subjects were enrolled initially. Three patients were excluded due to renal dysfunction and two were excluded due to revised diagnosis of CNV with AMD. Finally, a total of 119 patients with PCV and 119 matched controls were enrolled in this study.

The mean age was 72.1±13.0 years in the PCV group and 69.3±10.9 years in the control group, with a male preponderance (74.8%) in both groups. There were no statistically significant differences in age, gender, medical histories of hypertension, DM, coronary artery disease, cerebrovascular event and lifestyle factors including smoking and alcohol consumption between case subjects and controls (Table 1).

**Table 1:** Basic characteristics of patients with polypoidal choroidal vasculopathy and the control subjects.

	PCV (n = 119)	Control (n = 119)	P value
Age (years)	72.1±13.0 (36.0–93.0)	69.3±10.9 (36.0–94.0)	0.07
Gender (% male)	89 (74.8%)	89 (74.8%)	1.00
Medical history			
Hypertension	56 (47.1%)	54 (45.4%)	0.80
Diabetes mellitus	26 (21.8%)	28 (23.5%)	0.76
Coronary artery disease	21 (17.6%)	19 (16.0%)	0.73
Cerebrovascular event	5 (4.2%)	3 (2.5%)	0.47
Lifestyle determinants			
Smoking (current/former)	8 (6.7%)	4 (3.4%)	0.24
Alcohol consumption	2 (1.7%)	0 (0%)	0.16

PCV, polypoidal choroidal vasculopathy;

Continuous variables are presented as mean ± standard deviation (range) and compared using Student's *t*-test.

Categorical variables were presented as numbers (percentage) and compared using Pearson's chi-square test.

The median plasma homocysteine level was significantly higher in the PCV group (median, 12.20 µmol/L; interquartile range, 9.67–16.66 µmol/L) than in the control group (median, 9.80 µmol/L; interquartile range, 8.13–11.26 µmol/L; p<0.001). The homocysteine evaluation can be further categorized into subgroup with DM and without DM. The plasma homocsyteine level of PCV patients with DM was significantly higher than controls with DM (p = 0.001). Also, the plasma homocsyteine level of PCV patients without DM was significantly higher than controls without DM (p<0.001). The median serum hsCRP level was slightly higher in the PCV group (median, 0.16 mg/dl; interquartile range, 0.06–0.30 mg/dl) than in the control group (median, 0.11 mg/dl; interquartile range, 0.06–0.25 mg/dl, p = 0.07) (Table 2).

**Table 2:** Plasma homocysteine and serum C-reactive protein levels in overall and different genders of patients with polypoidal choroidal vasculopathy and the control subjects.

	Overall			Male			Female		
	PCV (n = 119)	Control (n = 119)	P value	PCV (n = 89)	Control (n = 89)	P value	PCV (n = 30)	Control (n = 30)	P value
Homocysteine (µmol/L)	12.20 (9.67–16.66)	9.80 (8.13–11.26)	<0.001	13.91 (11.43–17.65)	10.48 (8.86–11.91)	<0.001	9.29 (7.70–11.11)	8.18 (7.31–9.00)	0.02
hsCRP (mg/dl)^a^	0.16 (0.06–0.30)	0.11 (0.06–0.25)	0.07	0.17 (0.06–0.30)	0.11 (0.06–0.24)	0.14	0.15 (0.06–0.42)	0.09 (0.06–0.26)	0.22

hsCRP, high sensitivity C-reactive protein; PCV, polypoidal choroidal vasculopathy

Continuous variables are presented as median (interquartile range) and compared using the Mann-Whitney U test.

a Only 117 patients of PCV and 112 control subjects received examination of the serum hsCRP level.

The 95^th^ percentile of the homocysteine level in the control group was 13.26 µmol/L. Of the 119 PCV patients, 47 (39.5%) patients exceeded this cutoff value, compared with 5 of 119 (4.2%) patients in the control group (p<0.001). The 95^th^ percentile of the hsCRP level in the control group was 0.70 mg/dl. In the PCV group, 13 of 118 (11.1%) patients exceeded the cutoff value, compared with 6 of 113 (5.4%) patients in the control group (p = 0.12) (Table S1).

After stratified by genders, both males and females of the PCV group had significantly higher plasma homocysteine levels (p<0.001 in male and p = 0.02 in female) and proportions of hyperhomocysteinemia (p<0.001 in male and p = 0.02 in female), compared to those of the controls. The results of hsCRP levels were generally insignificant except for the higher proportions of elevated hsCRP in female (26.7% vs 3.4% in controls, p = 0.01) (Table 2 and Table S1). If we stratified the homocysteine levels into tertiles, significantly higher proportion of PCV patients had homocysteine levels in the highest tertile, compared to controls (67.2% vs. 32.8% in controls, p<0.001) (Table 3).

**Table 3:** Distribution of polypoidal choroidal vasculopathy cases and controls within tertiles of homocysteine and C-reactive protein levels.

	PCV (n = 119)	Control (n = 119)	Multivariable-adjusted OR (95% CI)^a^	P value
Tertile of homocysteine (µmol/L)				
1 (≤8.62)	16 (13.5%)	40 (33.6%)	1.00 (reference)	
2 (>8.62–10.81)	23 (19.3%)	40 (33.6%)	1.99 (0.83–4.77)	0.12
3 (>10.81)	80 (67.2%)	39 (32.8%)	8.84 (3.68–21.21)	<0.001
Tertile of hsCRP (mg/dl)^b^				
1 (<0.07)	34 (29.1%)	38 (33.9%)	1.00 (reference)	
2 (0.07–<0.17)	26 (22.2%)	36 (32.2%)	0.77 (0.38–1.55)	0.46
3 (≥0.17)	57 (48.7%)	38 (33.9%)	1.72 (0.91–3.24)	0.10

CI, confidence interval; hsCRP, high sensitivity C-reactive protein; OR, odds ratio; PCV, polypoidal choroidal vasculopathy

Categorical variables were presented as numbers (percentage).

a Adjusted for age, gender, hypertension, diabetes mellitus, coronary artery disease, cerebrovascular event, smoking and alcohol consumption.

b Only 117 patients of PCV and 112 control subjects received examination of the serum hsCRP level.

In the multivariable logistic regression analysis, after adjusting for age, gender, hypertension, DM, coronary artery disease, cerebrovascular event, smoking and alcohol consumption, it was observed that elevated plasma homocysteine levels were significantly associated with an increasing risk of PCV (OR, 1.54; 95% confidence interval (CI), 1.33–1.79, p<0.001), but not the hsCRP (OR, 1.82; 95% CI 0.61–5.45, p = 0.29). If we defined hyperhomocysteinemia and elevated hsCRP as levels above the 95th percentile of the control group, hyperhomocysteinemia is still significantly associated with PCV (OR, 22.04; 95% CI 7.05–68.92, p<0.001) in the multivariable logistic regression analysis, but not with the elevated hsCRP (OR, 2.45; 95% CI 0.75–7.94, p = 0.14). Moreover, people in the highest tertile of homocysteine had an approximately 9-fold increased risk of PCV (OR, 8.84; 95% CI 3.68–21.21, p<0.001) in the regression model (Table 3).

Of the 119 PCV patients, 17 (14.3%) had bilateral involvement, 49 (41.2%) had right-side involvement and 53 (44.5%) had left-side involvement. Comparing the bilateral involvement with unilateral involvement of PCV, no statistically significant differences in plasma homocysteine levels, serum hsCRP levels, age, gender, lifestyle factors, or medical histories were noted between the two groups.

Of the 136 eyes with PCV, PCV lesions were found in the extrafoveal area in 74 (54.4%) eyes, juxtafoveal in 51 (37.5%) eyes, subfoveal in 9 (6.6%) eyes and peripapillary in 2 (1.5%) eyes. Branching vascular network was noted in 130 (95.6%) eyes. Eighty-one (59.6%) eyes had serous pigment epithelial detachment (PED), 16 (11.8%) eyes had hemorrhagic PED, 51 (37.5%) eyes had subretinal fluid and 20 (14.7%) eyes had subretinal hemorrhage.

## Discussion

In our study, total plasma homocysteine levels were significantly higher in the PCV group than in the control group (median, 12.20 µmol/L vs 9.80 µmol/L in controls, p<0.001). On a multivariable logistic regression analysis, each 1 µmol/L increase of plasma homocysteine was associated with a 1.5-fold increase in the likelihood of having PCV (OR, 1.54; 95% CI, 1.33–1.79, p<0.001). Hyperhomocysteinemia, defined as levels above the 95th percentile of the control group, remained a relevant factor for PCV after regression analysis (OR, 22.04; 95% CI 7.05–68.92, p<0.001). The increase in risk was also significant for the highest tertile of homocysteine for PCV (OR, 8.84; 95% CI 3.68–21.21, p<0.001). Hyperhomocysteinemia was identified to be associated with PCV in this study. However, whether the plasma homocysteine contributes to direct toxicity on the pathogenesis of PCV or acts as a disease marker needs to be further determined.

The pathogenesis of PCV remains largely unknown. Possible association with systemic cardiovascular risk factors, such as hypertension, has been proposed [30]. The histopathology studies of PCV also showed inner choroidal vessel abnormalities with arteriosclerosis change [10]–[12], [31]. A significant proportion of PCV patients in our study had hypertension or coronary artery disease (47.1% and 17.6%, respectively), provided additional evidence of the association between PCV and arteriosclerosis. Homocysteine is a potent excitatory neurotransmitter that binds to the N-methyl-D-aspartate (NMDA) receptor and leads to oxidative stress, cytoplasmic calcium influx, cellular apoptosis, and endothelial dysfunction, which contributes to atherogenesis [32]. Given the observation that PCV was a choroidal vessel abnormality with arteriosclerosis change, the toxic effect of hyperhomocysteinemia on vessel may contribute to the pathogenesis of PCV.

Kondo et al [33] identify the elastin gene, a potent and specific regulator of the migration and proliferation of vascular smooth muscle cells, is a susceptible gene for PCV. The dysfunction of this signaling may lead to the development of vascular diseases such as arteriosclerosis [34]. The disruption of vascular elastin has also been proven to lead to aneurysm formation on animal model [35]. We proposed that elastin gene variation, which may be associated with the loss of function of vascular smooth muscle cells and the elastic layer of the vessel walls, together with homocysteine-mediated oxidative stress and endothelial dysfunction, and intraluminal pressure dysregulation, may lead to the final endpoint of choroidal aneurysmal bulging in PCV.

After stratified by gender, the plasma homocysteine level was found to be significantly different between male and female in our study (median, 13.91 umol/L in male with PCV vs. 9.29 umol/L in female with PCV, p<0.001). The trend was similar in both case and control subjects. After puberty, males have higher plasma homocysteine levels than females, with a gender difference in plasma homocysteine of approximately 2 µmol/L [24]. The male-female difference has been attributed to sex differences in muscle mass, circulating sex hormones, and it may possibly modify the contribution of the methylenetetrahydrofolate reductase (MTHFR) mutation to homocysteine concentrations [36]. In our study, there were only 30 females in the PCV group, and they had a significantly lower rate of coronary artery disease compared to the males (3.3% in female vs. 22.5% in male p = 0.02). This may suggest different contributions to the pathogenesis in females with PCV, but further study is required given the small female sample size in this study.

Kikuchi et al [23] have found a significantly increasing risk for the highest quartile of hsCRP for PCV in Japanese. However, we found no significant difference in serum hsCRP levels between the PCV and control groups in our study. Although a higher proportion of elevated hsCRP seemed to be observed in females with PCV (p = 0.01), the small sample size precluded further analysis. From a previous report, focal aggregates of chronic inflammatory cells have been shown in the PCV specimens [37]. The discrepancy in the study results of hsCRP in PCV may suggest the role of chronic inflammation in the pathogenesis of PCV.

There were some limitations in this study. First, it was a retrospective case-control study in a single medical center. Second, MTHFR polymorphism, serum cobalamin and folate levels were not checked, which might influence plasma homocysteine levels [24]. However, the subjects in this study were all well-nourished and people who had history of taking vitamin supplements were excluded to prevent their effect on plasma homocysteine levels. Third, the median plasma homocysteine level (12.20 µmol/L) in the PCV group was within the conventional definition of normal range (<15 µmol/L). However, plasma homocysteine level may be affected by many factors including age, gender, ethnicity, nutritional status, lifestyle, and disease [24]. Reference intervals should be established for different populations or even different laboratories [24]. Considering the relative small sample size and heterogeneous characteristics in this study, the reference upper-limit was calculated as level above the 95th percentile for presumed healthy individuals. If we used conventional definition of hyperhomocysteinemia (>15 µmol/L), 40 (33.6%) PCV patients still had elevated plasma homocysteine level, compared to 1 subject (0.8%) in the control group (p<0.001). The association still existed in the male group (43.8% in PCV vs 1.1% in controls, p<0.001) but not in the female group (3.3% in PCV vs 0% in controls, p = 0.31). After adjusting for age, gender, hypertension, DM, coronary artery disease, cerebrovascular event, smoking and alcohol consumption, conventional hyperhomocysteinemia (>15 µmol/L) is still significantly associated with PCV (OR, 64.55; 95% CI 9.79–567.73, p<0.001) in the multivariable logistic regression analysis.

In conclusion, our study showed hyperhomocysteinemia was significantly associated with PCV. The results also supported PCV might be related to arteriosclerosis. Further prospective controlled trials with large sample sizes are needed to improve our understanding of the role of hyperhomocysteinemia in PCV.

## Supporting Information

Table S1 **Proportions of hyperhomocysteinemia and elevated C-reactive protein levels in overall patients and different genders of patients with polypoidal choroidal vasculopathy and the control subjects.**
(DOC)
