# Supplementary material for: Hyperhomocysteinemia in Patients with Polypoidal Choroidal Vasculopathy: A Case Control Study
Source: PLoS One. 2014 Oct 22;9(10):e110818. doi: 10.1371/journal.pone.0110818 (PMC4206435; doi:10.1371/journal.pone.0110818)
Supplement: Table S1 — Proportions of hyperhomocysteinemia and elevated C-reactive protein levels in overall patients and different genders of patients with polypoidal choroidal vasculopathy and the control subjects. (DOC) [file pone.0110818.s001.doc]

**Table S1. Proportions of hyperhomocysteinemia and elevated C-reactive protein levels in overall patients and different genders of patients with polypoidal choroidal vasculopathy and the control subjects**

|  | Overall | | | Male | | | Female | | |
| --- | --- | --- | --- | --- | --- | --- | --- | --- | --- |
|  | PCV (n=119) | Control (n=119) | P value | PCV (n=89) | Control (n=89) | P value | PCV (n=30) | Control (n=30) | P value |
| Hyperhomocysteinemia^a^ | 47 (39.5%) | 5 (4.2%) | <0.001 | 45 (50.6%) | 4 (4.5%) | <0.001 | 7 (23.3%) | 1 (3.3%) | 0.02 |
| Elevated hsCRP^ab^ | 13 (11.1%) | 6 (5.4%) | 0.12 | 9 (10.3%) | 4 (4.8%) | 0.18 | 8 (26.7%) | 1 (3.4%) | 0.01 |
| hsCRP, high sensitivity C-reactive protein; PCV, polypoidal choroidal vasculopathy  Categorical variables were presented as numbers (percentage) and compared using Pearson's chi-square test.  ^a^Hyperhomocysteinemia (>13.26 μmol/L overall, >13.28 μmol/L in males and >11.26 μmol/L in females) and elevated hsCRP (>0.70 mg/dl overall, >0.75 mg/dl in males and >0.39 mg/dl in females) were defined as values above the 95^th^ percentile of the controls overall or in different genders.  ^b^Only 117 patients of PCV and 112 control subjects received examination of the serum hsCRP level. | | | | | | | | | |
